# Editorial: AI approach to the psychiatric diagnosis and prediction

**DOI:** 10.3389/fpsyt.2024.1387370

**Published:** 2024-05-23

**Authors:** Wenjing Gao, Long Lu, Xuntao Yin

**Affiliations:** ^1^ Department of Radiology, Guangzhou Women and Children’s Medical Center, Guangzhou Medical University, Guangzhou, China; ^2^ School of Information Management, Wuhan University, Wuhan, China

**Keywords:** deep learning, artificial intelligence, psychiatric disorders, machine learning, ChatGPT

## Introduction

Artificial intelligence (AI) has shown immense potential in the domain of medical research and clinical practice, particularly in the area of mental health. Traditional methods of diagnosing and treating psychiatric disorders have largely relied on clinicians’ observations and patients’ self-reports, hampered by a lack of objective evidence (1). Given the complexity of psychiatric illnesses and their profound impact on patients’ lives, treatment approaches demand high individualization and precision. AI, especially advanced tools like machine learning (ML) and natural language processing (NLP), by analyzing extensive clinical datasets to identify patterns of pathology, has opened new perspectives for the diagnosis and prediction of psychiatric disorders (2). The application of AI not only enhances the accuracy of diagnoses but also enables continuous monitoring of patients’ behavioral and emotional changes, predicting disease progression and treatment responses, thereby facilitating early intervention and personalized treatment. Additionally, AI can automate a multitude of routine tasks, significantly alleviating the workload of physicians, increasing work efficiency, and allowing them to focus more on patient care and treatment (3).

This Research Topic includes a series of research articles that apply AI methods to the diagnosis and prediction of psychiatric disorders. Lau et al. innovatively enhanced the accuracy of automatically assessing the severity of depression by applying prefix vectors and parameter-efficient tuning techniques to pre-trained large language models. Kellogg and Sadeh-Sharvit emphasized how the “Practical AI Augmentation” framework can effectively enhance the work efficiency of clinicians and the quality of patient care. It explored the potential applications of AI technology in key areas such as automation, patient engagement, and clinical decision support, while directly addressing the technical and ethical challenges that may arise during implementation, offering practical solutions. Kerz et al. made significant advancements in the field of psychiatric health detection based on linguistic behavior, successfully demonstrating how to enhance the interpretability of AI models without sacrificing predictive accuracy. By integrating a Bidirectional Long Short-Term Memory (BiLSTM) model with a set of human-interpretable features, along with employing a multi-task fusion learning framework and interpretability techniques, the research not only improved the accuracy of predictions for psychiatric disorders but also significantly enhanced their interpretability. The BiLSTM model is an advanced type of recurrent neural network that can capture long-term dependencies in both forward and reverse directions of a sequence, making it particularly suitable for tasks where context from both past and future is crucial. Hadar-Shoval et al. confirmed the ability of ChatGPT to describe the emotional responses of individuals with borderline personality disorder and schizophrenia, while Elyoseph and Levkovich revealed the limitations of ChatGPT compared to psychiatric health professionals in suicide risk assessment, particularly in evaluating patients’ risk of suicide attempts and psychological resilience. Although ChatGPT theoretically showed potential in psychiatric health assessments, practical outcomes indicated a tendency to underestimate suicide risks.

Despite the significant potential that AI demonstrates in the field of mental health, its practical deployment also is walking into a set of complex challenges. The most prominent issue is how to ensure the privacy and security of patient data when applying AI. This necessitates the joint development of strict data management and utilization standards by technology developers, medical professionals, and policymakers to protect sensitive patient information. Furthermore, enhancing the interpretability of AI models presents a significant challenge. The opacity of AI decision-making processes can affect the clinical acceptance. Therefore, ensuring that the decision-making processes of AI can be understood by both doctors and patients is critical. As we anticipate AI to bring revolutionary changes to the field of psychiatry, we must approach these technologies with cautious optimism. Interdisciplinary collaboration will be key to successfully integrating AI into clinical practice of psychiatric diagnosis and prediction.

Moreover, ensuring the reliability and validity of research results against known “ground truths” is indispensable. Initially, internal validation, which involves using a part of the same dataset for cross-validation within the study, can test the robustness of the models but may not fully reveal their performance on independent datasets. Therefore, external validation, which involves testing the accuracy of model predictions using independent datasets, is a critical step in confirming the broad applicability of the models. This method can assess the models’ generalizability, especially across different populations and geographic locations. However, acquiring high-quality, standardized, and independent datasets can be challenging in the field of mental health.

Considering these challenges, future research should focus on developing and implementing more rigorous validation mechanisms. This includes expanding the diversity of datasets to cover a broader range of demographic characteristics and promoting the creation of more publicly available benchmark datasets to enable thorough external validation. Simultaneously, improving the transparency and interpretability of models will help medical professionals and patients better understand the AI decision-making process, thus enhancing their acceptance and reliability in clinical practice.

## Author contributions

WG: Writing – original draft. LL: Writing – original draft. XY: Writing – review & editing.
